# Identification and Functional Analysis of the S-Layer Protein SplA of *Paenibacillus larvae*, the Causative Agent of American Foulbrood of Honey Bees

**DOI:** 10.1371/journal.ppat.1002716

**Published:** 2012-05-17

**Authors:** Lena Poppinga, Bettina Janesch, Anne Fünfhaus, Gerhard Sekot, Eva Garcia-Gonzalez, Gillian Hertlein, Kati Hedtke, Christina Schäffer, Elke Genersch

**Affiliations:** 1 Institute for Bee Research, Department of Molecular Bee Pathology, Hohen Neuendorf, Germany; 2 Department für NanoBiotechnologie, NanoGlycobiology, Universität für Bodenkultur Wien, Wien, Austria; Stanford University, United States of America

## Abstract

The Gram-positive, spore-forming bacterium *Paenibacillus larvae* is the etiological agent of American Foulbrood (AFB), a globally occurring, deathly epizootic of honey bee brood. AFB outbreaks are predominantly caused by two genotypes of *P. larvae*, ERIC I and ERIC II, with *P. larvae* ERIC II being the more virulent genotype on larval level. Recently, comparative proteome analyses have revealed that *P. larvae* ERIC II but not ERIC I might harbour a functional S-layer protein, named SplA. We here determine the genomic sequence of *spl*A in both genotypes and demonstrate by *in vitro* self-assembly studies of recombinant and purified SplA protein in combination with electron-microscopy that SplA is a true S-layer protein self-assembling into a square 2D lattice. The existence of a functional S-layer protein is novel for this bacterial species. For elucidating the biological function of *P. larvae* SplA, a genetic system for disruption of gene expression in this important honey bee pathogen was developed. Subsequent analyses of *in vivo* biological functions of SplA were based on comparing a wild-type strain of *P. larvae* ERIC II with the newly constructed s*pl*A-knockout mutant of this strain. Differences in cell and colony morphology suggest that SplA is a shape-determining factor. Marked differences between *P. larvae* ERIC II wild-type and mutant cells with regard to (i) adhesion to primary pupal midgut cells and (ii) larval mortality as measured in exposure bioassays corroborate the assumption that the S-layer of *P. larvae* ERIC II is an important virulence factor. Since SplA is the first functionally proven virulence factor for this species, our data extend the knowledge of the molecular differences between these two genotypes of *P. larvae* and contribute to explaining the observed differences in virulence. These results present an immense advancement in our understanding of *P. larvae* pathogenesis.

## Author Summary

*Paenibacillus larvae* is the most devastating bacterial pathogen of honey bees. However, the molecular interactions between infected larvae and *P. larvae* are poorly understood and little more than speculation exist concerning virulence factors. Recently, a putative S-layer protein has been identified in *P. larvae*. We here demonstrate that only representatives of *P. larvae* genotype ERIC II harbor a functional *spl*A-gene and that SplA is a true S-layer protein with self-assembly capability. The presence of a functional S-layer protein is novel for *P. larvae*. When elucidating the biological function of SplA we broke new ground by establishing primary cell culture for pupal gut cells and by developing a genetic system for disruption of gene expression in this important honey bee pathogen. By using these novel methods we were able to prove that SplA serves as a shape-determining factor, mediates adhesion to host cells, and is a key virulence factor of *P. larvae* ERIC II. These results present an immense advancement in our understanding of *P. larvae* pathogenesis. Furthermore, we propose *P. larvae* as a model system for the analysis of the *in vivo* functions of S-layer proteins because *P. larvae* SlpA knockout-mutants retain viability and are thus suitable for functional studies.

## Introduction

Due to their role as pollinators in natural and agricultural ecosystems, managed honey bees (*Apis mellifera*) are among the most important productive livestock [1], [2]. Therefore, pathogens and parasites attacking honey bees and causing fatal infectious diseases in individual bees which eventually might lead to the collapse of entire colonies have implications which reach far beyond apiculture [3], [4].

*Paenibacillus larvae* (*P. larvae*), a Gram-positive, rod-shaped, spore-forming bacterium, is the most devastating bacterial pathogen of honey bees. It is the etiological agent of the epizootic American Foulbrood (AFB), a non-rare, globally occurring brood disease which is classified as notifiable disease in most countries [5]. Only the spores of *P. larvae* are infectious and they can successfully establish an infection only in first instar larvae younger than 24–36 hours. Larvae become infected by consuming spore contaminated larval diet. Spores germinate in the midgut lumen where they massively proliferate before breaching the epithelium and invading the haemocoel [6]. Dead larvae are decomposed by *P. larvae* to a ropy mass. Lack of nutrients might then trigger sporulation of *P. larvae*. In the end, the ropy mass dries down to a hard scale which consists of billions of spores. These spores drive disease transmission within the colony as well as between colonies when either fed to young larvae or when distributed by contaminated adult bees. Hence, *P. larvae* is an obligate killer because transmission depends on the death of the host (for recent reviews see [7]–[9]).

Recently, different genotypes of *P. larvae* (*P. larvae* ERIC I–IV) have been described based on repetitive-element PCR using enterobacterial repetitive intergenic consensus (ERIC) primers [10] and it has been established that these genotypes differ phenotypically [5], [11], [12]. The most important phenotypic difference is the difference in virulence on larval level [13] which translates into virulence differences on colony level [14]. Infection bioassays with different strains of *P. larvae* representing the four genotypes ERIC I–IV revealed that *P. larvae* ERIC I strains needed about 13 days to kill all infected larvae (LT_100_ of about 13 days) while the other three genotypes ERIC II–IV turned out to be rather fast killers with an LT_100_ of about 5–7 days. Therefore, genotype ERIC I can be considered less virulent and the other three genotypes can be considered highly virulent on larval level. Several epidemiological studies revealed that only ERIC I and ERIC II are frequently isolated from AFB outbreaks worldwide [11], [15]–[17]. Therefore, these two genotypes are the most important genotypes in clinical honey bee pathology.

*P. larvae* undoubtedly is an important honey bee pathogen, therefore, it is enigmatic that the molecular pathogenesis of AFB still remains elusive. Proteases have been implicated as virulence factors since the early days of AFB research when it became evident that *P. larvae* secretes an astounding level of proteolytic activity [18], [19]. However, so far the exact role of proteases in the disease process could not be established and no specific protease could be identified as being involved in pathogenesis [20], [21]. Recently, enolase, an enzyme involved in sugar metabolism, has been presented as putative virulence factor although the possible mechanism of action remained elusive [22]. The existence of two different *P. larvae* genotypes (ERIC I and II) with opposing virulence and the availability of a draft genome sequence of *P. larvae* [23] enabled us to search for putative virulence factors via comparative genomics [24] and proteomics [25]. The latter approach led to the identification of a putative surface- (S-) layer protein which is expressed by *P. larvae* ERIC II but is missing from the proteome of *P. larvae* ERIC I [25]. S-layer (glyco)proteins, in general, are water-insoluble proteins endowed with an intrinsic capability to self-assemble into two-dimensional crystalline arrays *in vivo*, completely covering bacterial cell surfaces during all stages of the growth cycle, as well as *in vitro*, either in suspension or on several supports. With up to 15% of the total protein biosynthesis effort of a bacterial cell being devoted to S-layer protein synthesis, it is conceivable that the S-layer plays a pivotal role for the bacterium in its natural environment through providing a protective coat against external host or natural environmental forces as well as an adhesion and surface recognition mechanism [26], [27].

While discrete functions of S-layers still remain largely elusive, it is its predicted involvement in cell surface-associated phenomena that has triggered investigations on the role of S-layers in bacterial pathogenesis. *Bacillus anthracis*, for instance, synthesizes two S-layer proteins (Sap and EA1) [28] containing a ligase domain and an adhesin domain [29], which both are proposed to be of relevance for the establishment of anthrax [30]. *Clostridium difficile*, the etiological agent of antibiotic-associated diarrhea and pseudo-membraneous colitis [31], expresses two superimposed S-layers, which were shown to be involved in the mechanism of gut colonization and in the process of adhesion to the intestinal mucosa [32], [33] as well as in modulation of the function of human monocytes and dendritic cells [34]. Other examples for S-layers acting as virulence factors concern the human pathogens *Tannerella forsythia* [35], [36], *Campylobacter rectus* [37] or *Campylobacter fetus* [38]. Among bacteria not affecting humans is *Aeromonas salmonicida* causing furunculosis in fish and *Bacillus thuringiensis*, an entomopathogenic member of the *B. cereus* group, with a broad host range among invertebrates. For both bacteria the contribution of the S-layer protein to pathogenesis could be demonstrated [39], [40].

The present study deals with the assessment of the virulence potential of a putative S-layer on the honey bee pathogen *P. larvae* ERIC II. Based on the availability of the naturally occurring S-layer deficient genotype ERIC I of *P. larvae* and the concomitant construction of a *P. larvae* ERIC II *slp*A-knockout (ko) mutant, a functional analysis of the SlpA S-layer was performed. More specifically, this study addresses the questions (i) whether a functional *spl*A gene exists in the genome of ERIC II but not of ERIC I, by using comparative genome analysis; (ii) whether the SplA protein of *P. larvae* ERIC II is a true S-layer protein, by performing *in vitro* self-assembly studies of purified, recombinant protein followed by electron microscopy analysis, and (iii) whether the putative S-layer protein SplA of *P. larvae* ERIC II has biological functions in pathogenesis. The latter question was addressed by comparing an *spl*A-ko-mutant with wild-type bacteria with respect to adhesion to larval midgut cells and mortality of infected larvae. These data demonstrated that SplA is a key virulence factor of *P. larvae* ERIC II. Furthermore, it is the first functionally proven virulence factor for the species of *P. larvae*, presenting an immense advancement in our understanding of *P. larvae* pathogenesis.

## Materials and Methods

### Bacterial strains and growth conditions

Several *P. larvae* field isolates [13] representing the *P. larvae* genotypes ERIC I and ERIC II [5], were used in the course of this study (Table 1). Most strains had been extensively characterized in previous studies [5], [13], [14], [25]. Genetic manipulation of *P. larvae* ERIC II resulting in the generation of S-layer knockout clones was performed with the field strain *P. larvae* 04-309 (DSM 25430), which had been shown to be a rather virulent strain on larval level with an LC_50_ of less than 100 cfu/ml for exposed larvae in previous studies [13]. Cultivation of the non-manipulated wild-type bacteria was performed as described previously [11], [12]. *P. larvae* 04-309 manipulated knockout clones were cultivated on MYPGP-agar plates [41] supplemented with 5 µg/ml chloramphenicol and incubated at 37°C for 2–3 days. For pre-cultures, a single bacterial colony was inoculated into 3 ml of MYPGP broth [41] supplemented with 5 µg/ml chloramphenicol and cells were grown for 16 h at 37°C with shaking (200 rpm). Subsequently, 10 ml of MYPGP broth supplemented with 5 µg/ml chloramphenicol were inoculated with a maximum of 300 µl of the pre-culture to adjust an OD_600_ = 0.01 and incubated at 37°C with shaking (200 rpm) until the early exponential phase was reached.

**Table 1:** Bacterial strains used in this study.

Strain	Source	ERIC genotype
ATCC 9545**	ATCC	I
02-075**	Honey (dis.col.)	I
02-081	Honey (dis.col.)	I
02-129**	Honey (dis.col.)	I
02-179	Honey (dis.col.)	I
02-250	Honey (dis.col.)	I
03-019	Honey (dis.col.)	I
03-119**	Honey (dis.col.)	I
03-122	Honey (dis.col.)	I
03-159**	Honey (dis.col.)	I
03-189** (DSM 25429)	Honey (dis.col.)	I
00-897**	Honey (dis.col.)	I I
00-1163**	Honey (dis.col.)	I I
01-1714	Honey (dis.col.)	I I
03-016**	Honey (dis.col.)	I I
03-194	Honey (dis.col.)	I I
03-195**	Honey (dis.col.)	I I
03-200**	Honey (dis.col.)	I I
03-478	Honey (dis.col.)	I I
03-518**	Honey (dis.col.)	I I
03-525** (DSM 16116)	Honey (dis.col.)	I I
04-309** (DSM 25430)	Honey (dis.col.)	I I

Note:

**: , strains characterized in [5], [12], [13], [25]; dis. col., diseased colony.

*Escherichia coli* DH5α cells (Invitrogen) transformed with plasmids pTT_*wsf*A243 [42] or pTT_*spl*A101 (see below) were cultivated in selective Luria Bertani (LB) media (agar and broth) supplemented with 30 µg/ml chloramphenicol. Plasmid DNA preparations were carried out following the manufacturer's protocols (QIAprep Spin Miniprep kit, Qiagen). Concentration and purity of the plasmid preparations were analyzed by photometric analysis (Nanodrop) and agarose gelelectrophoresis.

Preparation of spore suspensions for adhesion assays and exposure bioassays was performed as described previously [5], [13], [14]. Briefly, for sporulation of *P. larvae*, 100 *P. larvae* colonies resuspended in 300 µl brain heart infusion broth (Oxoid, Germany) were used to inoculate the liquid part of Columbia sheep blood agar slants followed by incubation at 37°C for 10 days. Subsequently, the liquid part was analyzed by phase contrast microscopy (VWR IT 400) for the absence of vegetative cells. Spore concentrations were determined by cultivating serial dilutions on Columbia sheep blood agar plates as described previously [11], [12].

Cell morphology of the S-layer knockout mutant *P. larvae* 04-309 Δ*spl*A and the parent wild-type strain *P. larvae* 04-309 was examined by scanning electron microscopy (SEM) (Phillips SEM 515). For this purpose, cells were grown in MYPGP broth at 37°C with shaking until the exponential phase was reached, harvested by centrifugation, and washed three times with sterile double-distilled water. Bacterial suspensions were deposited on an SEM grid and dried at room temperature. Dry grids were coated with gold in a sputter coater and analyzed using a scanning electron microscope operated at 15 kV.

### Determination of the SplA sequence in ERIC I and ERIC II strains

For determining the genomic sequence of the recently identified, putative S-layer protein of *P. larvae* ERIC II, we performed a TBLASTN analysis [43] with the obtained putative S-layer protein sequence of *P. larvae* 04-309 (ERIC II) [25] against the sequence of *P. larvae* BRL 230010 [23] accessed through Baylor *Paenibacillus larvae* Data (http://www.hgsc.bcm.tmc.edu/bcm/blast/microbialblast.cgi?organism=Plarvae). The best hit was with an open reading frame located in contig 240 and showing some homologies (e-value 9e-45, 47%) to the Sap (surface array protein) precursor of *B. anthracis* (ZP_02394181.1) but lacking SLH (S-layer homology)-domains. Next, we selected a primer pair (Table 2) with one primer (SplA-R5) located in the vicinity of the 3′-end of the sequence putatively coding for the S-layer SAP precursor and the other primer (SplA-F4) located upstream of the 5′-end of a neighbouring open reading frame exhibiting putative SLH domain sequences. The obtained amplicons of about 3166 bp in *P. larvae* 04-309 (ERIC II) and *P. larvae* 03-189 (ERIC I; DSM 25429) were sequenced (Eurofins MWG GmbH, Ebersberg, Germany) and analyzed. To verify that the genomic sequences obtained from *P. larvae* 04-309 (ERIC II) and *P. larvae* 03-189 (ERIC I) were not strain- but genotype-specific we sequenced the putative *spl*A-gene in another ten strains per genotype (Table 1). All sequence analyses were performed at Eurofins MWG GmbH (Ebersberg, Germany) and DNA alignments were performed by using the software Vector NTI (Invitrogen).

**Table 2:** Primers used for sequence analysis of *spl*A, screening of *P. larvae* isolates for *spl*A, gene knockout in *P. larvae* 04-309, and recombinant production of SplA.

primer name	primer sequences	used for
SplA-F1	5′-ATGAGGAATATGGGCTCCG-3′	sequencing
SplA-R1	5′-CTGTTTTTTCGTTAAGCATGGTT-3′	
SplA-F2	5′-ACTATCAGCAAATCGTTATTGAAGG-3′	
SplA-R2	5′-TCAACTGTTGTTGCACCGG-3′	
SplA-F3	5′-TATTAAACCTGGAAAAGTAGATGTCC-3′	
SplA-R3	5′-TTAAAGGTTTTTAACAAGATTACCAGC-3′	
SplA-F4	5′-GGTTAGCCAATATTGAAGCTCTG-3′	
SplA-R4	5′-AATCCGCAGAACCTTTAGCA-3′	
SplA-F5	5′-AAGATTTAATTGAAACTCTTAATGCAG-3′	
SplA-R5	5′-TCGGTACTGCAGAAAACTTTG-3′	
SplA-F	5′-ATATGGGCTCCGCTATTTCC-3′	screening
SplA-R	5′-AATCCGCAGAACCTTTAGCA-3′	
Slp773_NcoI-for	5′-aatcaCCATGGCGAGGAATATGGGCTCCGCTATTTCC-3′	recombinant production
Slp773_XhoI-re	5′-aatcaCTCGAGTTAAAGGTTTTTAACAAGATTACCAG-3′	
IBS_*splA*_101	5′-AAAAAAGCTTATAATTATCCTTAATAGCCCTGGAAGTGCGCCCAGATAGGGTG-3′	*spl*A knockout
EBS1d_*splA*_101	5-′CAGATTGTACAAATGTGGTGATAACAGATAAGTCCTGGAAACTAACTTACCTTTCTTTGT-3′	
EBS2_*splA*_101	5′-TGAACGCAAGTTTCTAATTTCGGTTGCTATCCGATAGAGGAAAGTGTCT-3′	

### Recombinant production of SplA

The sequence encoding the S-layer protein SplA of *P. larvae* 04-309 (ERIC II) was amplified from genomic DNA by PCR with the primer pair Slp773_NcoI-for and Slp773_XhoI-re (Table 2) (Invitrogen) using Phusion High-Fidelity DNA Polymerase (Fermentas) with annealing at 58°C and extension at 72°C for 80 s. PCR was performed in a thermal cycler My Cycler apparatus (Bio-Rad). The *spl*A amplification product was purified using the GeneJET Gel Extraction Kit (Fermentas), followed by digestion with NcoI/XhoI and insertion into the linearized and dephosphorylated expression vector pET28a(+) (Novagen). The resulting plasmid for was named pET28a-Spl773.

For overexpression of SplA, *E. coli* BL21Star (DE3) cells were transformed with pET28a-Spl773 by electroporation according to the manufacturer's instructions (Invitrogen). Subsequently, freshly transformed cells were grown in LB medium at 37°C and 200 rpm [44] to the mid exponential growth phase (corresponding to an OD_600_ of ∼0.6), protein expression was induced with a final concentration of 0.5 mM isopropyl-β-D-thiogalactopyranosid, and cultures were grown for additional 4 h at 37°C and 200 rpm, with kanamycin added to the medium at a final concentration of 50 µg/ml.

*E. coli* BL21 cells expressing SplA were harvested by centrifugation at 5,000×*g* (Beckman J2-Hs centrifuge and JA-10 rotor) at 4°C for 15 min. The pellet was resuspended in 10 ml of incubation buffer (50 mM Tris/HCl pH 7.2, 10 mM MgCl_2_, 0.5% Triton) per gram of wet pellet. Subsequently, 0.8 mg/ml of lysozyme (Sigma Aldrich) and 500 units of benzonase (Merck) per g of wet pellet were added and the suspension was incubated at 37°C for 30 min. 0.72 g of urea were added per ml of suspension, and buffer was added to obtain a final concentration of 6 M urea. Extraction was performed at 25°C with shaking for 30 min. Cellular debris and insoluble components were removed by centrifugation at 5,000×*g* (4°C, 15 min) followed by ultracentrifugation at 250,000×*g* (Beckman Coulter Optima L-100 XP Ultracentrifuge and 70Ti rotor) at 4°C for one hour. The supernatant was concentrated using Amicon Ultra centrifugal filters (Millipore) and subsequently applied to chromatography on a Superdex 200 prep grade XK16 FPLC-column (1.6×60 cm; GE-Healthcare, Uppsala, Sweden) using 50 mM Tris/HCl pH 7.2, containing 10 mM MgCl_2_ and 6 M urea, as eluent, and collecting 1-ml fractions at a flow rate of 1 ml/min. Fractions containing the SplA protein according to Coomassie-stained SDS-PAGE were pooled and dialyzed at 4°C against distilled water containing 10 mM CaCl_2_ to promote self-assembly.

### Transmission electron microscopy of negatively-stained SplA protein

An approximately 1 mg/ml-suspension of SplA protein was applied to Formvar and carbon coated 300-mesh TEM grids (Agar Scientific) that were rendered hydrophilic upon glow discharge using a Pelco easiGlow apparatus (Ted Pella). The grids were incubated for 3 min face down on a drop of the protein suspension. Samples were fixed with 2.5% glutaraldehyde, washed three times with MilliQ water, and stained with 1% uranyl acetate solution (pH 4.2) for 150 s [45]. Samples were investigated using a Tecnai G2 20 Twin transmission electron microscope (TEM; FEI), operating at 80 keV. Pictures were taken with an FEI Eagle 4 k CCD camera (4096×4096 pixels). The magnification was calibrated by using negatively stained catalase crystals [46]. Image processing and lattice refinement were done with software developed in house based on Fourier domain techniques [47], [48].

### Gene knockout

To specifically create a vector for the knockout of *spl*A in *P. larvae* 04-309 (ERIC II), we used vector pTT_*wsf*A243 [42]. This vector derived from vector pTT_*wsf*P1176 [49], targeted for intron insertion at position 243/244 from the start codon of *wsf*A in *Paenibacillus alvei*. WsfA and WsfP both play a role in S-layer glycosylation reactions in *P. alvei* [42]. The vector is a fusion of the commercially available targetron vector pJIR750ai (Sigma) offering the bacterial mobile group II intron LI.LtrB sequence of *Lactococcus lactis*, the *Geobacillus*-*Bacillus*-*E.coli* shuttle vector pNW33N, and the *sgs*E S-layer gene promoter of *G. stearothermophilus* NRS 2004/3a [50] upstream of the intron cassette.

Specific disruption of the *spl*A-gene in *P. larvae* 04-309 (ERIC II) was performed following a recently described strategy [49]. The LI.LtrB targetron of vector pTT_*wsf*A243 was retargeted prior to transformation into *P. larvae* 04-309 (ERIC II). For this purpose, identification of potential insertion sites of the intron in the *spl*A open reading frame and design of PCR primers for the modification of the intron RNA was accomplished by a computer algorithm (http://www.sigma-genosys.com/targetron). Modified intron RNA sequences (EBS1, EBS2, δ) specifically base pair with the insertion site target sequences in *spl*A (EBS1, EBS2, δ′), leading to intron insertion and disruption of the gene. The algorithm predicted 25 possible insertion sites, with position 101 (position 101 from the initial start codon of *spl*A) having the highest predicted insertion efficiency (E-value 0.004). Three primers (IBS *spl*A 101, EBS1d *spl*A 101, and EBS2 *spl*A 101, Table 2) were necessary to retarget the intron to base pair with the *spl*A target site sequence in the *P. larvae* chromosome. The retargeted Ll.LtrB targetron (353 bp) was subsequently digested with restriction enzymes *Hind*III and *Bsp*1407I (FastDigest, Fermentas) and ligated into pTT_*wsf*A243 digested with the same restriction enzymes, thereby replacing the *wsf*A targetron and generating the plasmid pTT_*spl*A101. The mutant *P. larvae* 04-309 strain was analyzed for the presence of intron DNA in *spl*A and presence/absence of SplA in 2D-SDS-PA gel electrophoresis as described recently [25].

### Creation of *P. larvae* 04-309 (ERIC II) knockout mutants

Electrocompetent *P. larvae* 04-309 cells (ERIC II) were prepared as described [51] and 1 µg of plasmid pTT_*spl*A101 was transformed by electroporation as recently established [52]. Cells were recovered in MYPGP broth for 16 h, plated out on MYPGP-agar containing 5 µg/ml chloramphenicol and incubated for 3 days. Clones were screened for intron insertion by PCR analysis using primers flanking the SplA intron insertion position 101 of the ORF: SplA-F and SplA-R (Table 2). *Spl*A knockout was further verified by sequencing the PCR product of *P. larvae* 04-309 Δ*spl*A. Colony morphology of the knockout mutant *P. larvae* 04-309 Δ*spl*A and the parent wild-type strain *P. larvae* 04-309 was evaluated on both, CSA and MYPGP agar plates after cultivation at 37°C for 3 days. Growth rates of both strains were assessed in liquid broth (MYPGP) by measuring the OD_600_ until the stationary phase was reached. Cell shapes of both strains were also examined by using a scanning electron microscopy (see above).

### Adhesion assays

For adhesion assays, pupal gut cells were isolated from 10 days-old pupae. Pupae were shortly immersed in H_2_O_2_ and washed with 1×PBS (137 mM NaCl, 2.7 mM KCl, 10 mM Na_2_HPO_4_, 2 mM KH_2_PO_4_; pH 7.4). Heads were cut off; thorax and abdomen were fixed on a Sylgard coated dish and carefully opened. Cold L15 medium (Sigma-Aldrich) containing 10% antibiotic-antimycotic solution (Sigma-Aldrich) was prepared as described before (Kreissl and Bicker, 1992) and carefully added on the dish. The guts were extracted and transferred into a well of a 24-well plate with fresh L15 medium pre-warmed at 37°C; up to ten guts were put into one well. The medium was carefully removed and 1 ml of enzyme solution was added (0.05% trypsin and 0.5% collagenase/dispase). The plate was incubated with gentle shaking at 4°C, 30°C and 4°C for 1 hour each. Subsequently, guts in medium were transferred to a 1.5 ml-reaction tube and centrifuged for 3 minutes with 300×*g*. The supernatant was discarded and the pellet was gently homogenized with 40 µl of L15 medium (pre-warmed at 37°C) per gut. 40 µl of cell suspension were dispensed per well of a 96-well plate, and after 20 minutes of incubation 60 µl of pre-warmed (37°C) BM3 medium [53] were added per well. After 24 hours incubation at 33°C, the medium was discarded and replaced with 100 µl fresh BM3 medium. Cells were cultured for 6 or 7 days before used in adhesion assays. Medium was exchanged every second day. Vitality of the cells was checked using Invitrogen's Mitotracker Red FM protocol to specifically label active mitochondria in live cells following the manufacturer's instructions: Gut cells were incubated in chamber slides, washed with BM3 medium and adhered cells were incubated with 100 µl BM3 medium supplemented with Mitotracker Red FM in a final working concentration of 300 nM at 33°C for 1 h. Subsequently, the supernatant was removed and cells were washed three times with 100 µl of 1×PBS. Supernatant was removed and cells were fixed with 100 µl Roti Histofix (Roth) for 20 minutes at room temperature. Subsequently, cells were washed with 1×PBS. For visualisation of the nuclei, cells were additionally treated with 1 µg/ml DAPI solution (in methanol, Roche) for 10 minutes. Supernatant was removed and cells were intensely washed with 1×PBS. The chamber was removed and cells were embedded with ProLong gold antifade reagent (Invitrogen) before fluorescence activity detection (Nikon Ti-E Inverted Microscope). Pupal gut cells were analyzed microscopically using DIC for light microscopy, the TexRed filter for detection of active mitochondria and the DAPI filter for nucleus visualisation.

Adhesion assays were performed according to a modified protocol developed for *Streptococcus pyogenes* [54]. Briefly, bacterial cells were grown as 5 ml-cultures in MYPGP medium to the early exponential phase (OD_600_ 0.3). Bacterial cultures were centrifuged at 4°C for 10 min at 3214×*g* and the pellet of one 5 ml-culture was resuspended in 5 ml of pre-warmed (37°C) BM3 cell medium. A 96 well plate containing 6 to 7 day old monolayers of pupal gut cells was inoculated with bacterial suspensions. 100 µl of bacterial suspension was added to each well and incubated at 37°C for 1 hour. Wells were extensively washed 3 times with 1×PBS to remove unattached bacteria. For counting colony forming units (CFU), eukaryotic cells were detached and lysed for 10 min at 37°C with 50 µl of 0.5% trypsin per well. Subsequently, cell-adherent bacteria were plated for enumeration. Three dilutions of each well were plated and incubated for 3 days at 37°C. The average number of bacteria recovered per well was determined from three independent wells. Experiments were repeated three times and the percentage of adherent bacteria was calculated in relation to *P. larvae* 04-309 wild-type adherent bacteria (100%) in each experiment. Data were analyzed by one-way analysis of variance (ANOVA) followed by the Bonferroni post-hoc test using GraphPad Prism 5 software (***p-value<0.001).

### Exposure bioassays

To analyse the functional role of SplA during pathogenesis, exposure bioassays were performed essentially as already described [5], [13]. First instar larvae of the infection groups were exposed to larval diet (66% royal jelly (v/v), 33% glucose (w/v) and 33% fructose (w/v)) containing spores of *P. larvae* 04-309 wild-type (ERIC II) and of the SplA deficient knockout mutant *P. larvae* 04-309 Δ*spl*A at a final concentration of 100 cfu/ml (corresponding to about LC_80_ of the wild-type strain to ensure that observation of both, decrease and increase in mortality would be possible). A concentration of 100 cfu/ml will result in about 1 cfu theoretically taken up by each larva assuming that first instar larvae consume about 10 µl larval diet during the experimental time window for infection (24 hours). An infection dose of 1 cfu per larva is the lowest reasonable dose and, hence, concentrations of less than 100 cfu/ml were not tested. Control larvae were fed with normal larval diet throughout the entire experiments. An experiment consisted of three replicates of 30 larvae each: one infected with the knockout mutant *P. larvae* 04-309 Δ*spl*A, one infected with the parent wild-type strain *P. larvae* 04-309, and one non-infected control. Each experiment was performed three times (n = 3) and each time larval health status and mortality were monitored daily over 15 days. Dead animals were classified as dead from AFB only when vegetative *P. larvae* could be cultivated from the larval remains. Surviving animals were also analyzed for the presence of vegetative *P. larvae*. On no occasion was *P. larvae* cultivated from remains of dead control animals or from survivors (pupae at day 15 post infection) of any of the three replicates. SplA knockout stability was proven by PCR with the gene specific primer pair SplA-F and SplA-R, flanking the intron insertion site 101. PCR amplicons were analyzed by gel electrophoresis on a 1% agarose gel, stained with ethidium bromide and visualised by UV light.

Total mortality of the *P. larvae* 04-309 wild-type and the *P. larvae* Δ*spl*A mutant was calculated for each replicate as proportion of larvae that died from AFB compared to the total number of exposed larvae. Data represent mean values ± SEM. To obtain the time course of infection and to calculate the LT_100_ (lethal time, i.e., time it takes the pathogen to kill all infected animals, [55]), the cumulative proportion of AFB-dead larvae per day (100% equals all AFB-dead larvae in this replicate) was calculated for each replicate and plotted against time [5], [13]. Survivors were excluded from this calculation because the LT is a measure for the proportion of dead animals only [55]. Data represent mean values ± SD of three independent infection assays, each with three replicates and 30 larvae per replicate; data were statistically analyzed with an unpaired Student's T Test using GraphPad Prism 5 software.

### Accession numbers

The GenBank accession numbers for *spl*A of *P. larvae* ERIC I and ERIC II are JQ353715 and JQ353714, respectively.

## Results

### Determination and analysis of the sequence of the putative S-layer protein gene *spl*A of *P. larvae* ERIC II

**Figure 1:**
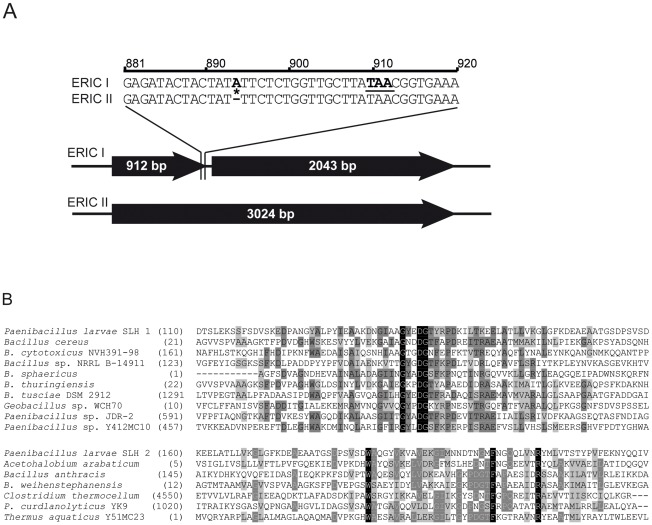
Analysis of the genomic and protein sequence of *P. larvae* SplA. (A) In contrast to *P. larvae* ERIC II representatives, all *P. larvae* ERIC I strains investigated showed an additional adenin at position 894 (bold and highlighted by a star), leading to a frameshift mutation causing a premature translational stop at TAA (bold and underlined). This obviously results in the observed lack of a functional SplA in representatives of *P. larvae* ERIC I. (B) For SLH-domain prediction of SplA the CDD (Conserved domains and protein classification, NCBI) was applied. Two predicted domains with homology to SLH domains of other *Bacillaceae* (*B. anthracis*, *B. weihenstephanensis*) but also to SLH domains of other genera were identified. *P. larvae* SLH 1 domain of SplA (upper panel) includes residues 117–164 of SplA and exhibits homologies to several SLH domains of *Bacillaceae*. *P. larvae* SLH 2 domain of SplA (lower panel) includes residues 188–220 and exhibits homologies not only to other *Bacillaceae* but also to SLH domains of other genera. Identical amino acids are marked in black, conserved amino acids are highlighted in dark grey and blocks of similar residues are presented with a light grey background. Strains showing homologous domains are listed alphabetically.

By comparative proteome analysis between *P. larvae* ERIC I and ERIC II we recently demonstrated the expression of a putative S-layer protein SplA in ERIC II but not in ERIC I strains of *P. larvae* [25]. To verify this difference on genome level we first determined the sequence of the corresponding gene in several ERIC I and ERIC II strains (Table 1). Sequence analysis revealed that both genotypes harbor a gene putatively coding for an S-layer protein. However, in *P. larvae* ERIC I strains, the gene was interrupted by a frameshift mutation due to an inserted adenine (A) resulting in a premature stop of translation due to a stop codon TAA; the insertion and resulting frameshift mutation was missing in *P. larvae* ERIC II strains (Fig. 1A). Therefore, only in *P. larvae* ERIC II a functional gene for a putative S-layer protein could be identified. Protein BLAST alignment of the translated ERIC II sequences with translated sequences of S-layer protein genes from other *Bacillaceae* revealed that the *P. larvae* ERIC II protein harbors all conserved domains characteristic of S-layer proteins. Using the Conserved Domain Database (CDD; NCBI) two regions in the N-terminal part of the protein coding for SLH (S-layer homology) domains belonging to SLH superfamilies [56]–[58] were identified. These two domains are predicted to range from residues 117–164 (SLH1) and 188–220 (SLH2) (Fig. 1B).

### Self-assembly studies with the recombinant protein

To support the S-layer protein status of the putative *P. larvae* ERIC II SplA protein self-assembly studies were performed with the recombinant protein. SplA was expressed from pET28a-Spl773 in *E. coli* BL21 cells and subsequently purified from 4-h cultures by gel permeation chromatography with 6 M urea contained in the eluent, which was necessary to keep the proteins disintegrated (*i.e*., in the monomeric state). Fractions 26–29 containing the S-layer protein according to SDS-PAGE (not shown) were pooled. The molecular mass of rSplA on the SDS-PA gel corresponded to the calculated molecular mass of 113.5 kDa (not shown). After 3 hours of dialysis of the S-layer pool against 10 mM CaCl_2_ solution, typical self-assembly products were visible in the dialysis tube. The dialysate was diluted to a protein concentration of approximately 1 mg/ml and subjected to negative-staining.

**Figure 2:**
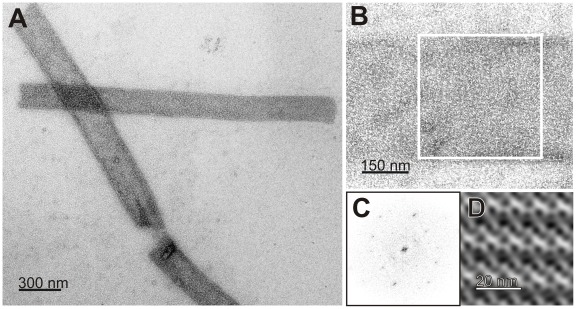
TEM micrographs of negatively stained self-assembly products of recombinant purified S-layer protein SplA. (A) Cylindrical self-assembly products of the recombinant purified S-layer protein SplA of *P. larvae* 04-309 (ERIC II); (B) Enlarged region of a cylinder showing the regular lattice; (C) Power spectrum of the S-layer patch indicated in (B); (D) S-layer lattice reconstruction.

The TEM micrographs of negatively-stained rSplA clearly revealed the formation of monolayered, cylindrical self-assembly products with dimensions of approximately 2 µm length and 180 nm width (Fig. 2A). Within these cylinders, the S-layer protein species are aligned in a 2D lattice of good long-range order (Fig. 2B–D) exhibiting lattice parameters of approximately 10.0 nm×15.4 nm and γ = 90°. These lattice parameters are in good agreement with those of the S-layer lattices from diverse members of the *Bacillaceae* family [59].

### Construction of a *P. larvae* SplA-knockout-mutant (04-309 Δ*spl*A)

**Figure 3:**
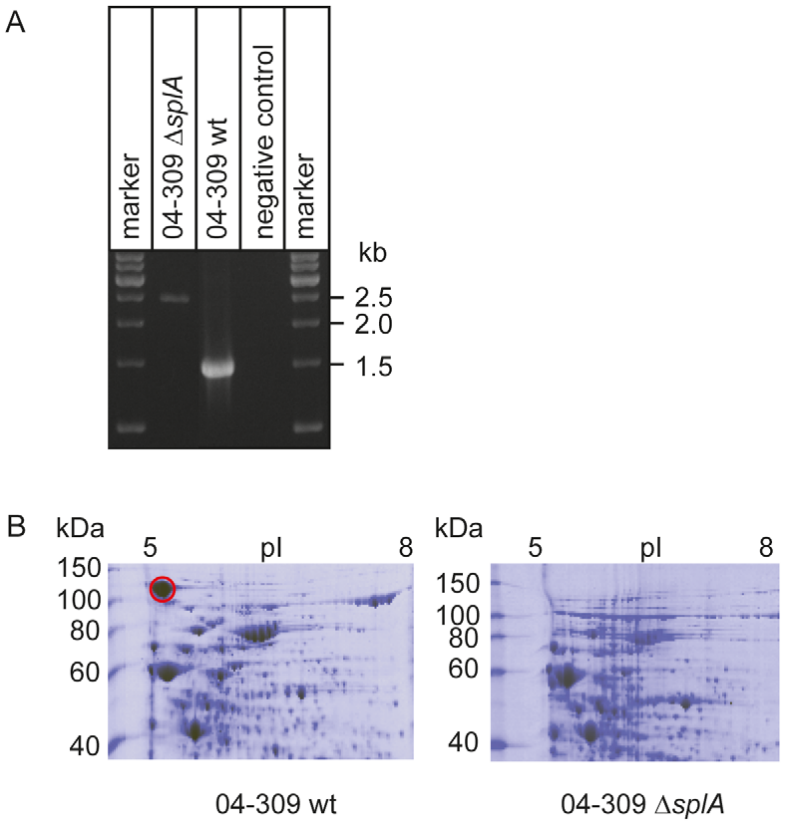
Verification of the knockout of *spl*A in 04-309 (ERIC II) on genome and proteome level. (A) Verification of the successful knockout of *spl*A in *P. larvae* 04-309 (ERIC II) by the targetron knockout system is demonstrated on genome level. Primers flanking insertion site 101 in *spl*A amplify a 1530 bp product in the wild-type (04-309 wt), whereas the amplicon of the mutant *P. larvae* strain (04-309 Δ*spl*A) migrated at 2445 bp as expected. (B) Proteome analyses of *P. larvae* knockout mutant 04-309 Δ*spl*A and the parent wild-type *P. larvae* strain (04-309 wt) is shown. Cytosolic proteins extracted from bacteria in the stationary growth phase were separated by 2D-SDS-PAGE and stained with PageBlue (Fermentas). The protein patterns differed significantly in the occurrence of the highlighted protein spot (red circle) recently identified as a putative S-layer protein by mass spectrometry. The conspicuous spot of the *P. larvae* 04-309 wild-type was lacking in the knockout mutant *P. larvae* 04-309 Δ*spl*A.

**Figure 4:**
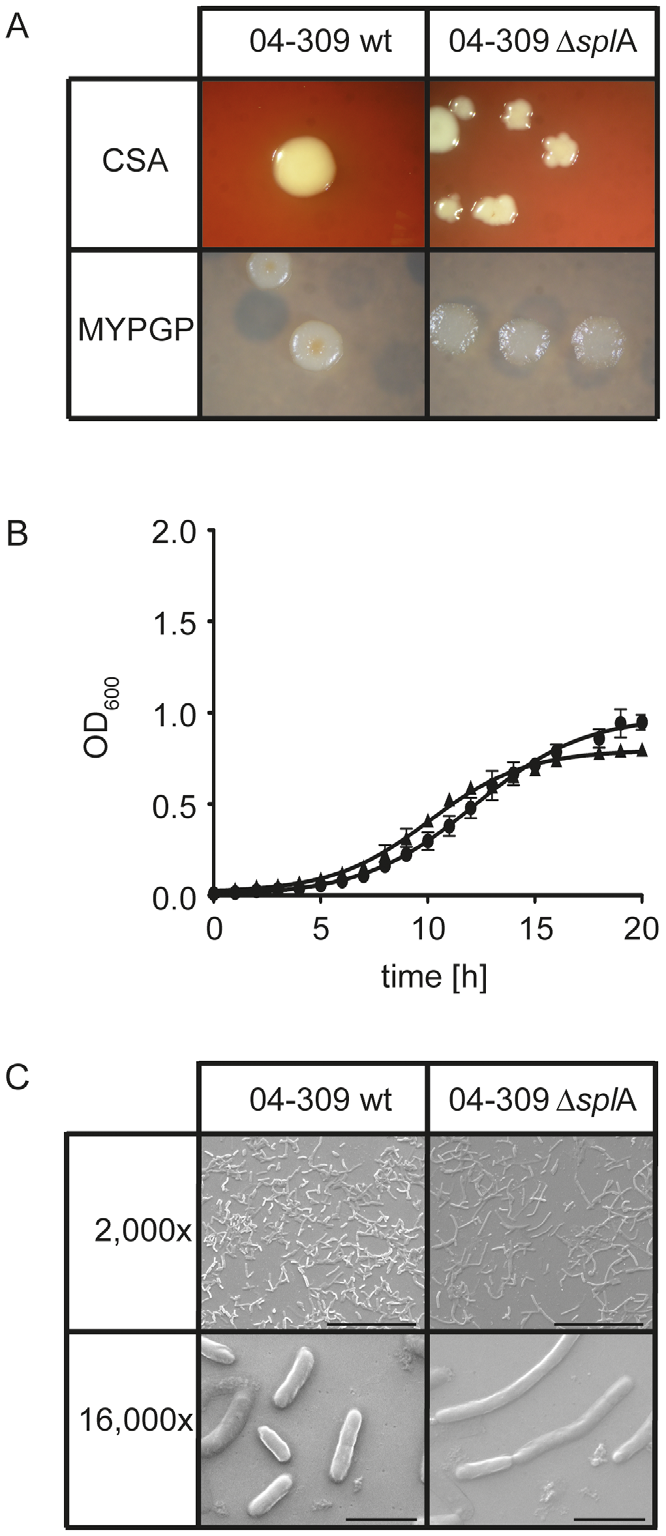
Analysis of *P. larvae* 04-309 versus *P. larvae* 04-309 Δ*spl*A. (A) Differences in colony morphology of the *spl*A knockout mutant *P. larvae* 04-309 Δ*spl*A on CSA and MYPGP agar in comparison to the parent wild-type strain *P. larvae* 04-309 were evident. (B) Growth of wild-type strain *P. larvae* 04-309 (black squares) and the *spl*A knockout mutant *P. larvae* 04-309 Δ*spl*A (black triangles) in liquid broth did not differ significantly (p-value = 0.878; unpaired Student's t-test). (C) Electron microscopy analysis revealed differences in cell morphology between the S-layer knockout mutant *P. larvae* 04-309 Δ*spl*A and the parent wild-type strain *P. larvae* 04-309. The mutant cells were rather elongated and assembled to longer chains than the wild-type cells. Bars represent 50 µm (2,000 fold) and 5 µm (16,000 fold).

To be able to functionally analyze SplA, *P. larvae* ERIC II mutants deficient in the expression of SplA were constructed. This was accomplished by using the commercially available Targetron System (Sigma-Aldrich) with a modified vector [49] which leads to gene disruption due to the insertion of an intron into the target gene. This may be a less elegant method then, e.g., homologous recombination leading to gene deletion, however, it is the first method ever developed for knocking out gene expression in *P. larvae* and it was the only method that worked with this bacterium. Successful insertion of the *spl*A-specific retargeted intron (915 bp) into the target gene *spl*A of the *P. larvae* ERIC II strain 04-309 was demonstrated by PCR-analysis of the corresponding genomic region. While the wild-type amplicon had the expected size of 1530 bp, the mutant amplicon carrying the insertion migrated at 2445 bp (Fig. 3A). This insertion resulted in loss of expression of SplA in the knockout-strain, designated *P. larvae* 04-309 Δ*spl*A, as demonstrated by 2D-SDS-PAGE analysis (Fig. 3B). The dominant spot for SplA in the SDS-PA gel of the parent wild-type strain is totally absent from the gel of the knockout mutant strain. Further analysis showed that the mutant strain *P. larvae* 04-309 Δ*spl*A clearly differed from the wild-type strain in colony morphology both on Columbia sheepblood agar (CSA) plates as well as on MYPGP agar plates (Fig. 4A). On CSA plates the mutant *P. larvae* 04-309 Δ*spl*A no longer showed the usual circular colony form but, instead, the colonies grew with an irregular shape indicative of irregular growth on these plates. Such irregularities were not observed on MYPGP-plates. On these plates the colonies of the mutant strain just showed a different color and opacity indicating that growth of the mutant bacteria was not generally impaired. Accordingly, growth of the mutant *P. larvae* 04-309 Δ*spl*A and the parent wild-type strain in liquid broth did not differ significantly (p-value = 0.878; data were analyzed with an unpaired Student's t-test using GraphPad Prism 5 software) (Fig. 4B) and germination and sporulation of both strains were also similar (data not shown).

By scanning electron microscopy (SEM) analysis, we observed that the vegetative cells of 04-309 Δ*spl*A were elongated and resembled more the cells of *P. larvae* ERIC I (data not shown) while the wild-type strain *P. larvae* 04-309 exhibited a rather short and stubby cell morphology (Fig. 4C).

### Functional studies with the wild-type and the mutant strains of *P. larvae* 04-309 (ERIC II)

**Figure 5:**
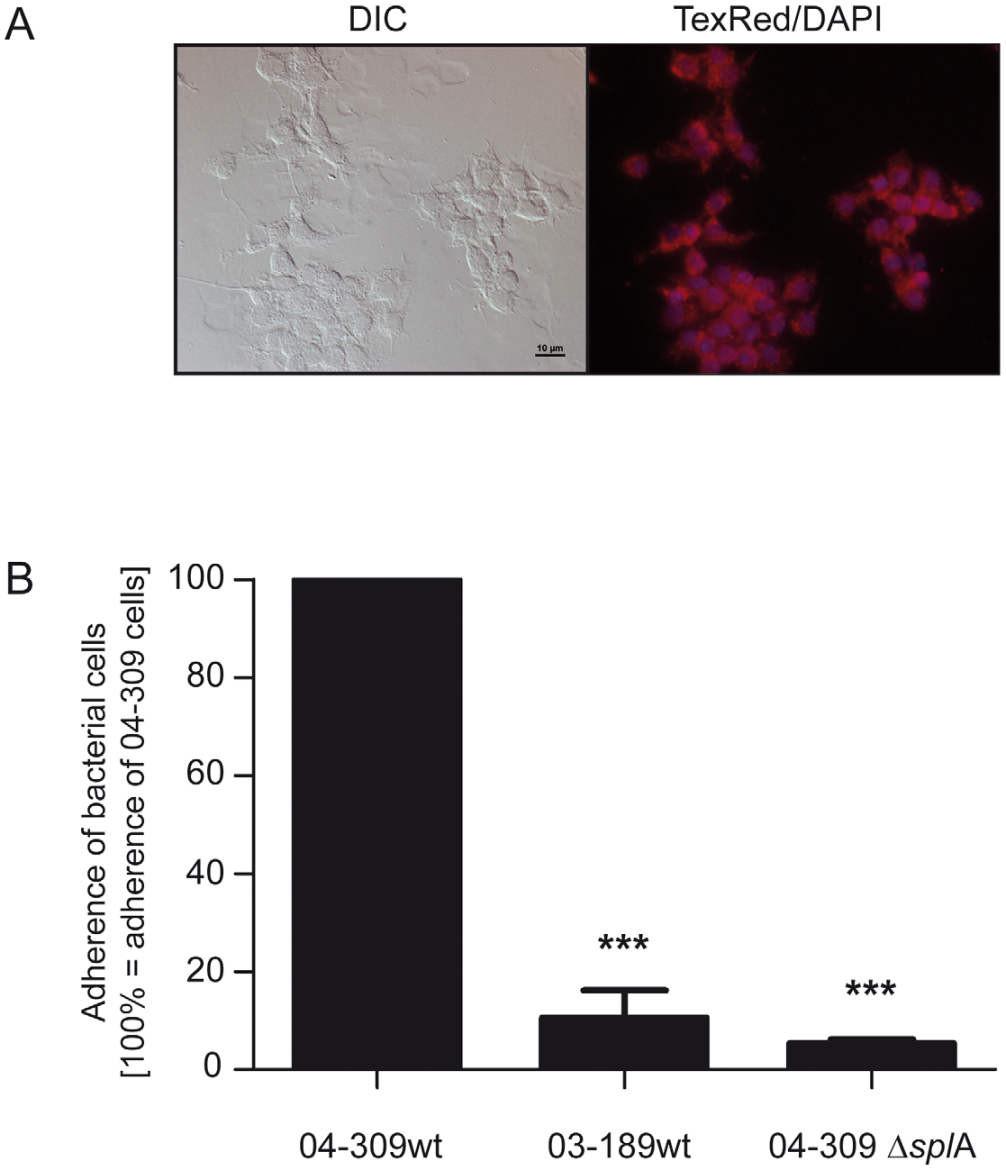
Adhesion of *P. larvae* 04-309 Δ*spl*A and *P. larvae* 04-309 wild-type to primary pupal gut cells. (A) Vitality of primary pupal gut cells after 6 days of cultivation was analyzed using Mitotracker Red FM (Invitrogen) to specifically label active mitochondria indicative for live cells. Cells were microscopically analyzed using Differential Interference Contrast (DIC) (left) as well as TexRed (mitochondria) and DAPI (nuclei) filters to visualize fluorescence signals (right). (B) Percentage of *P. larvae* 03-189 wild-type (03-189 wt; ERIC I, naturally SplA-deficient) and *P. larvae* 04-309 Δ*spl*A (SplA knockout mutant) associated to pupal gut cells after 60 min of incubation, extensive washing, and cell lysis; results are presented as mean ± SEM of three independent-experiments and are related to cell-associated bacteria of the wild-type strain *P. larvae* 04-309 (04-309 wt; ERIC II), i.e. the amount of *P. larvae* 04-309 cell-associated bacteria in each experiment equalled 100%. Data were analyzed by one-way analysis of variance (ANOVA) followed by the Bonferroni post-hoc test (***p<0.001).

We hypothesized that SplA might be involved in or mediate bacterial adhesion to midgut cells. To test this we performed cell adhesion assays with *P. larvae* 04-309 Δ*spl*A and the parent wild-type strain *P. larvae* 04-309 as well as *P. larvae* 03-189, representing the naturally S-layer deficient genotype *P. larvae* ERIC I [25] using primary pupal midgut cells (Fig. 5A). Comparing the cell adhesion capacity of the two wild-type strains *P. larvae* 04-309 and *P. larvae* 03-189 representing the two different genotypes ERIC II and ERIC I, respectively, revealed a significantly lower adhesion of the ERIC I strain versus the ERIC II strain: The cell adhesion capacity of *P. larvae* 03-189 was only 10.59%±5.61% (mean ± SEM) of the adhesion capacity of *P. larvae* 04-309 (*p*-value 0.001). Comparing the wild-type strain *P. larvae* 04-309 with the mutant strain *P. larvae* 04-309 Δ*spl*A gave a similar result: The *spl*A-knockout mutant had a residual adhesion of as little as 5.36%±0.86% (mean ± SEM) compared to the parent wild-type strain (Fig. 5B) (*p*-value 0.001). These results strongly support a function of SplA in bacterial adhesion to the larval midgut.

**Figure 6:**
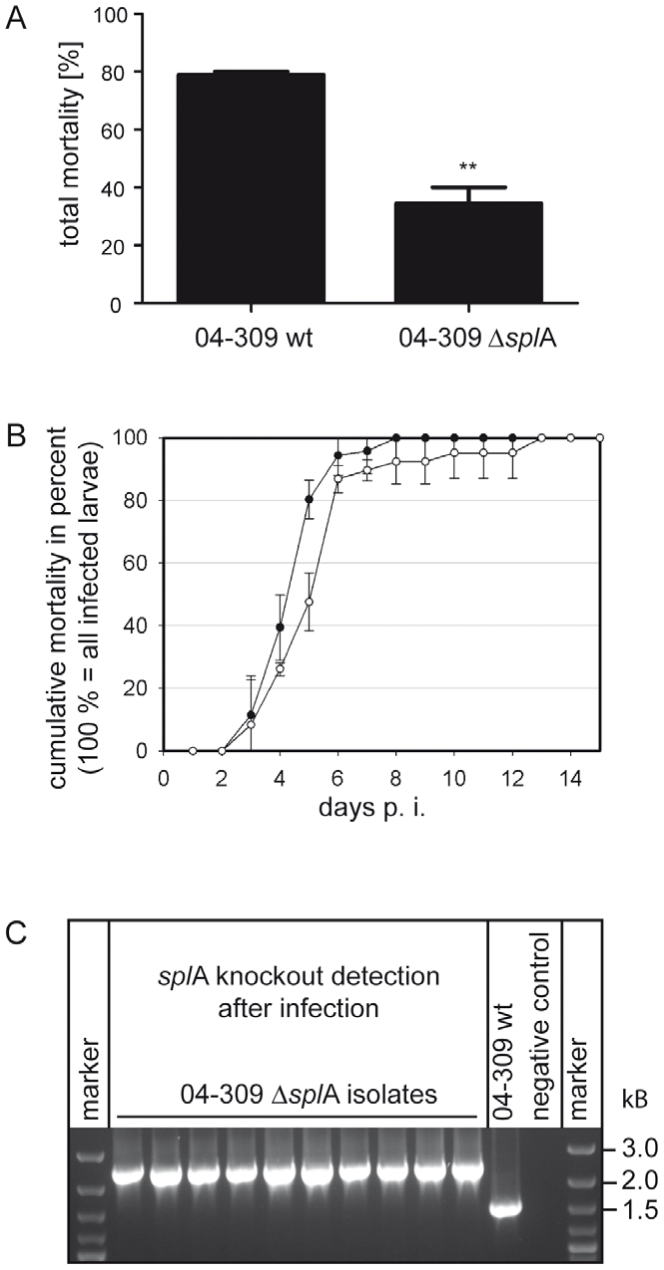
Analysis of the virulence potential of SplA using exposure bioassays. (A) Total mortality of *P. larvae* 04-309 Δ*spl*A and the parent wild-type strain *P. larvae* 04-309 was assessed in exposure bioassays. Larvae infected with the SplA-knockout mutant showed a significantly decreased mortality (paired T Test, p-value = 0.0098) although the same amount of spores was fed. Data represent mean values ± SEM of three independent infection assays. (B) To obtain the time course of infection and the LT (lethal time), cumulative mortality of *P. larvae* 04-309 Δ*spl*A (open circles) and the parent wild-type strain *P. larvae* 04-309 (filled circles) was assessed in exposure bioassays and plotted against time post infection (days). 100% represented all larvae that died from *P. larvae* infection during the course of the experiment. No significant difference between both strains in the time course of infection could be observed (p-value = 0.6767; data represent mean values ± SD of three independent exposure bioassays and were analyzed by an unpaired Student's T Test). (C) To evaluate the stability of the knockout mutation and to verify that bacteria killing the larvae in the mutant groups still carried the mutation *P. larvae* isolated from dead larvae of the mutant groups were analyzed for the presence of the intron cassette. The amplicon of the knockout mutant *P. larvae* 04-309 Δ*spl*A is about 900 bp longer than the amplicon from the wild-type *P. larvae* strain (04-309 wt). Representative results for ten isolates are shown.

Successful adhesion to the larval midgut might be an important step in pathogenesis. We therefore performed exposure bioassays in the laboratory with *P. larvae* 04-309 Δ*spl*A and *P. larvae* 04-309 wild-type. Groups of first instar larvae were either fed with spores of the wild-type strain or with spores of the mutant strain both at a concentration of 100 cfu/ml. This spore concentration resulted in ∼80% mortality in the *P. larvae* wild-type infected larvae corroborating our previous results which showed an LC_50_ of less than 100 cfu/ml for this strain [13]. Control larvae were mock infected and used as internal quality control of the exposure bioassays [13]. Each dead larva of the infected groups was analyzed and only those larvae which died from *P. larvae* infection were included in the calculation. Comparing larval mortality in the groups infected with wild-type bacteria and mutant bacteria revealed a significant ∼55% decrease in larval mortality in the larvae infected with *P. larvae* 04-309 Δ*spl*A: While the wild-type strain killed 78.9%±1.13% of the larvae, the mutant strain killed only 34.4%±5.55% of the exposed larvae (Fig. 6A). Because *P. larvae* 04-309 Δ*spl*A was still able to kill larvae, SplA does not appear to be an essential factor for pathogenicity. However, the virulence of the mutant was markedly reduced demonstrating that SplA is an important virulence factor.

Representatives of the *P. larvae* genotype ERIC II are characterized by killing the larvae rather fast with an LT_100_ of about 6–7 days as opposed to the LT_100_ of ERIC I strains which is about 12 days [7], [8], [13]. To unravel whether SplA is involved in influencing the time course of disease we determined the LT_100_ of the mutant and the wild-type parent strain and analyzed the time course of disease by cumulatively calculating the proportion of larvae that died from AFB in both groups (Fig. 6B). The time course of infection did not differ significantly between the parent and the mutant strain (p-value = 0.6767; data were analyzed with an unpaired Student's t-test using GraphPad Prism 5 software). The LT_100_ of the wild-type strain was 7.0±1.0 (mean ± SD) days confirming previous data [13]. The LT_100_ of the mutant strain did not differ significantly with 10.3±2.5 (mean ± SD) days (p-value = 0.214). In both groups about 90% of the infected animals died before the onset of metamorphosis, *i.e.* on day 6 to 7 post infection in the exposure bioassays (Abb. 6B) [7], [8]. These results indicate that SplA is not involved in determining the time course of disease or the rather low LT_100_ of *P. larvae* ERIC II when compared to *P. larvae* ERIC I. All bacteria isolated from dead larvae were analyzed via PCR to verify their identity as *P. larvae*. Bacteria isolated from *P. larvae* which died from 04-309 Δ*spl*A infection were additionally analyzed via PCR and it was confirmed that they still carried the insertion (Fig. 6C).

## Discussion

Despite being one of the most important honey bee pathogens, the pathogenesis of *P. larvae* infections is poorly understood hampering the development of sustainable control or curative measures. The existence of different genotypes of *P. larvae* which differ in virulence [5], [13] opened the possibility to explore the virulence mechanisms by simply comparing these genotypes using different –omics approaches. Comparative genomics using suppression subtractive hybridization (SSH) [60] allowed us to identify several putative virulence factors including toxins and secondary metabolites [24]. Recently, comparative proteome analysis led to the identification of an S-layer protein SplA expressed only by the highly virulent genotype *P. larvae* ERIC II but missing in representatives of *P. larvae* ERIC I [25]. Considering the role of S-layer proteins in several pathogenic bacteria it was self-evident to hypothesize that *P. larvae* SplA is involved in the infection process. However, studies on the role of distinct proteins in the molecular pathogenesis of *P. larvae* infections in honey bee larvae have been hampered in the past by the lack of a genetic system allowing the targeted disruption of gene expression in this pathogen. Recently, a gene knockout system for application in *Paenibacillus alvei* has been published [49] which proved to be applicable to *P. larvae*, too. Using this system we were able to knock out the SplA gene in *P. larvae* and to analyze the biological function of SplA because *P. larvae* knockout-mutants for SplA turned out to be viable and could be used for functional studies, especially infection studies. Thus, we here provide a most valuable molecular tool and previously unavailable means for studying *P. larvae*, especially *P. larvae* pathogenesis. Analyzing the *in vivo*-function of specific bacterial proteins will now be possible which will greatly enhance our understanding of the interaction between *P. larvae* and honey bee larvae. In addition, *P. larvae* can serve as a model system to analyze the *in vivo* function of an S-layer protein.

The *P. larvae* ERIC II S-layer protein SplA possesses all characteristics of a typical, functional S-layer protein. The protein stretches over 1008 amino acids, including a predicted signal sequence comprised of 49 amino acid residues at the N-terminal part of the protein. The N-terminal signal sequence is proposed to be cleaved off after position 49 (predicted by SignalP 4.0 server: [61]). The mature protein without the signal sequence has a predicted molecular weight of around 103.5 kDa and an isoelectric point of 4.97. SplA possesses two S-layer homology (SLH) domains as predicted by CDD (NCBI: [56], [57]). These features predict this protein to target the bacterial surface via the secretory pathway during cell proliferation [59], [62]–[64]. SplA exhibits strong homology to an S-layer protein Sap precursor of *B. anthracis* and it also has homology to S-layer proteins from other bacteria, like *B. weihenstephanensis* (Sap: ADQ08578, 74% query coverage, e-value 1e-67) and *Geobacillus sp.* WCH70 (S-layer protein: YP_002951037, 81% query coverage, e-value 9e-84). In addition, studies with the recombinant SplA of *P. larvae* confirmed that the proposed SplA protein is a true S-layer protein fully covering the bacterial cell surface and capable of self-assembling. These features are essential in the context of analyzing the functional role of the S-layer of *P. larvae*.

Pathogenesis can be divided into three steps: After encountering a host, firstly, the pathogen needs to enter the host, secondly, it has to establish infection and thirdly, it will cause disease [55]. None of these steps is remotely understood for *P. larvae*. Recently, it was shown that the life cycle of *P. larvae* in infected larvae starts with a non-invasive phase in which *P. larvae* massively proliferates in the midgut lumen obviously without affecting the midgut epithelium [6]. During this stage of infection *P. larvae* has not yet entered the host because the midgut lumen is still considered ‘outside’. Entering the host is accomplished not until the bacteria breach the epithelium. For breaching the epithelium bacteria might need to adhere to the host cells followed by invasion into host tissue. Many bacterial structures attached to or protruding from the cell surface mediate host cell adhesion as prerequisite for invasion. S-layer proteins are known bacterial adhesins and it was this capacity to mediate adhesion to host cells that led to research into the role of S-layer proteins in pathogenesis. And indeed, in distinct pathogenic bacteria it could be demonstrated that S-layer proteins or proteins containing SLH-domains are key factors in pathogenesis.

For instance, for the S-layer proteins of *Tannerella forsythia*, an important bacterium in periodontitis, it was recently shown that they are responsible for adhesion to host cells. Impaired adhesion in the S-layer deletion mutants resulted in reduced invasion of the host cells. However, no difference in virulence between the wild-type strain and the deletion mutants could be observed [35]. In the present work, we demonstrated that the S-layer protein SplA of *P. larvae* mediated adhesion to host cells. Loss of SplA in the knockout-mutant resulted in loss of bacterial adhesion to larval midgut cells. Adhesion of *P. larvae* to the midgut epithelial cells might be a prerequisite for the subsequent breaching of the epithelium and invasion of the haemocoel which was shown to be accompanied by larval death [6]. We, therefore, speculated that larval mortality would be lower in the knockout-mutant due to impaired adhesion capacity. And indeed, in exposure bioassay a significant reduction of larval mortality in the larvae infected by the S-layer knockout-mutant compared to the parent wild-type strain could be observed. These results suggest that SplA mediated adhesion of *P. larvae* ERIC II to the larval midgut epithelium plays an important role in the adhesion to and breaching of the epithelium, which are both considered key steps in the pathogenesis of *P. larvae* infections [7]. Although the factors involved in the subsequent invasion process still remain elusive we have shed some light onto the first step in the molecular pathogenesis of *P. larvae* ERIC II infections. An additional role of SplA in the recognition by *P. larvae* immune receptors or in escaping the immune system of the larvae cannot be excluded and will be addressed in future studies.

Interestingly, *P. larvae* ERIC I does not possess a functional *spl*A-gene and does not express an S-layer protein although it is as lethal as *P. larvae* ERIC II albeit less virulent [13]. This suggests that *P. larvae* ERIC I has developed a different strategy to accomplish invasion of the haemocoel and killing the larva. It is noteworthy in this context that by suppression subtractive hybridization we could identify several toxins in ERIC I but not in ERIC II [24] also suggesting that these two genotypes do not only differ in virulence but presumably also differ in their pathogenicity mechanisms. Obviously there are several ways to kill a larva in the course of AFB and it will be most interesting to elucidate the different strategies.

In addition to their proposed role in virulence, S-layer proteins were shown to play an important role in maintaining cell shape and structure [65]. Comparing colony morphology and cell shape between the parent and the mutant *P. larvae* strain revealed that lack of expression of SplA in 04-309 Δ*spl*A indeed led to a change in cell and colony morphology (Fig. 4). Therefore, SplA not only is an important factor during infection, it also determines cells shape and colony morphology of *P. larvae*. In *B. anthracis*, similar morphological differences between S-layer deficient mutants and wild-type parent strains have been observed [66]: *B. anthracis* S-layer knockout mutants show a lengthened cell shape and a change in colony morphology.

The present work provides convincing evidence that SplA is a key virulence factor of *P. larvae* ERIC II involved in the early steps of pathogenesis, i.e. in the adhesion of the bacteria to larval midgut cells. These findings are a major progress in our understanding of the molecular pathogenesis of *P. larvae*. In addition, this study clearly advances our understanding of *in vivo* functions of S-layers. Interestingly, S-layer knockout constructions have not been described for *Lactobacilli* and *Paenibacilli* so far, since in these bacterial species S-layer proteins obviously play an obligate role in cell maintenance [67], [68]. Since *P. larvae* is one of the few examples where SplA knockout-mutants are viable, we propose *P. larvae* as a model organism for *in vivo*-studies of S-layer protein function.
